# Effect of Olfactory Stimulus on the Flight Course of a Honeybee, *Apis mellifera*, in a Wind Tunnel

**DOI:** 10.3390/insects5010092

**Published:** 2013-12-31

**Authors:** Hidetoshi Ikeno, Tadaaki Akamatsu, Yuji Hasegawa, Hiroyuki Ai

**Affiliations:** 1 School of Human Science and Environment, 1-1-12 Shinzaike-Honcho, Himeji-shi, Hyogo 670-0092, Japan; E-Mail: eisen_drgoon@yahoo.co.jp; 2 Honda Research Institute Japan Co. Ltd., Wako-shi, Saitama 351-0188, Japan; E-Mail: yuji.hasegawa@jp.honda-ri.com; 3 Division of Biology, Department of Earth System Science, Fukuoka University, 8-19-1 Nanakuma, Jonan-ku, Fukuoka 814-0180, Japan; E-Mail: ai@fukuoka-u.ac.jp

**Keywords:** odor source search, flight control, foraging flight, honeybee, wind tunnel

## Abstract

It is known that the honeybee, *Apis mellifera*, uses olfactory stimulus as important information for orienting to food sources. Several studies on olfactory-induced orientation flight have been conducted in wind tunnels and in the field. From these studies, optical sensing is used as the main information with the addition of olfactory signals and the navigational course followed by these sensory information. However, it is not clear how olfactory information is reflected in the navigation of flight. In this study, we analyzed the detailed properties of flight when oriented to an odor source in a wind tunnel. We recorded flying bees with a video camera to analyze the flight area, speed, angular velocity and trajectory. After bees were trained to be attracted to a feeder, the flight trajectories with or without the olfactory stimulus located upwind of the feeder were compared. The results showed that honeybees flew back and forth in the proximity of the odor source, and the search range corresponded approximately to the odor spread area. It was also shown that the angular velocity was different inside and outside the odor spread area, and trajectories tended to be bent or curved just outside the area.

## 1. Introduction

It has been shown that a honeybee can remember the scent of a flower and mark it using pheromones during foraging [1,2]. As the odor caused by incoming nectar affects various activities [3,4,5], such as food processing and forager recruiting in the nest, it must have a crucial role in the foraging process. Studies have been carried out on how insects use odor signals for orientation in activities, such as foraging and mating. Behaviors when approaching upwind of the odor source have been established for various insects, such as ants, flies and moths [6,7,8,9,10,11,12,13,14,15]. In particular, some moth species can approach pheromone sources using a zigzag flight [7,8,10]. In the case of the silkworm moth, programmed sequential behavior was observed with the acquisition of a pheromone, that is, walk straight, zigzag-turn and then loop, by sensing a pulse of pheromone. A newly sensed pheromone resets this process [16].

In the case of honeybees, it has been difficult to establish the flight control properties induced specifically by the odor stimulus, because they navigate over a wide area at high speed using various sources of information, such as color and the shape of flowers. To trace their flight locus, a radar-tracking system was applied by attaching an antenna to their thorax. It was revealed from the experiments that a honeybee would approach an odor source from the windward side using Lévy flight, which is a mixture of straight flights and random directional changes [17]. However, flight courses in the field were strongly dependent on the conditions, such as visual features, wind direction, and so on [18]. These experiments were mainly focused on the global properties of flights over a wide area and did not have the precision to analyze local changes in a flight route caused by odor acquisition. There was also the problem of removing the effect of odor spreading in the field. Flight experiments in a restricted area could be useful for observing, in detail, the processes for sensing an odor and controlling a flight course.

In this study, honeybee flights were recorded in a wind tunnel when visiting an odor source. The trajectories of flight were extracted and analyzed to reveal the effect of odor on flight course. It was shown that a honeybee switched the goal of its flight from the memorized position from the training phase to the odor-emitting position in the test phase.

## 2. Materials and Methods

### 2.1. Honeybee

The honeybee, *Apis mellifera*, from a single hive kept on the Himeji-Kankyoningen campus of the University of Hyogo, 1-1-12 Shinzaike-Honcho, Himeji-shi, Hyogo 670-0092, Japan, was used throughout all our experiments. The hive was located 5 m from the entrance of a wind tunnel for behavioral observation. Foragers in the hive were guided into the tunnel and collected food in the middle of the tunnel as part of their daily tasks. The honeybees were trained to come to the feeder in the tunnel as their daily foraging activity. Filter paper with 50% sucrose water and 5 mL of pure orange oil (Wako chemical, Osaka, Japan) was used for the food source.

### 2.2. Wind Tunnel

**Figure 1:**
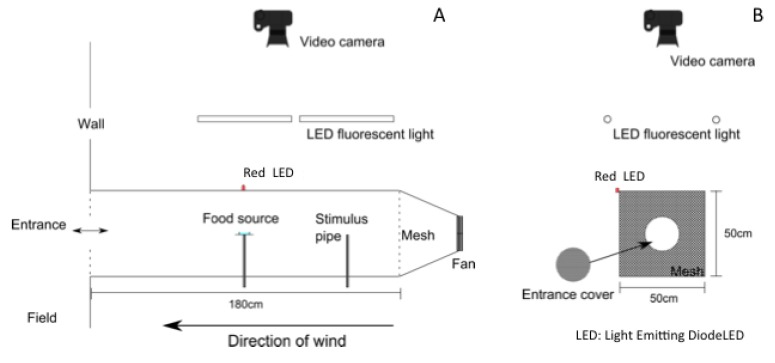
(**A**) Side view of the wind tunnel; (**B**) entrance of the tunnel.

A wind tunnel (50 cm high, 50 cm wide and 180 cm long) was used for the flight space (Figure 1). The floorboard and sideboard of the tunnel were made out of white colored plywood without any landmarks. An acrylic board covered the ceiling of the tunnel from which the honeybee flight was recorded. Blackout curtains covered the ceiling and windows of the room to provide a uniform visual stimulus along the wind tunnel. The entrance wall of the tunnel was connected to the outside wall of the room and covered by a sheet with 1 mm mesh. An entrance hole 15 cm in diameter was located at the center of the entrance wall for entering and leaving the tunnel. 

Four wind fans (D8025B12M, Elan Vital, Ping Jen, Taiwan) were located on the other side of the entrance wall to generate a wind flow from inside to outside the tunnel. Wind flow speed was kept at less than 0.1 m/s during the experiments. A mesh screen was set in front of the fans to prevent bees from entering the fan area. Two metal pipes (2 mm diameter, 25 cm high) were located in the center (the feeding position) and 50 cm upwind of the center. The tunnel was illuminated by four parallel white Light Emitting Diode (LED) lights (Lumichal FL13L06K50A, N-hitech, Gimpo, Korea). 

A video camera (Exilim F1, Casio, Tokyo, Japan) for recording the honeybees’ flight was positioned above the tunnel. The recording area started about 30 cm from the entrance. The recorded images were at 640 × 480 pixels resolution and 30 fps. To record the timing of the stimulus in the video, a red LED light was lit when the odor stimulus was turned on.

### 2.3. Odor Stimuli

Throughout the experiments, orange oil was used for the odor source. Small pieces of filter paper with and without 5 mL of pure orange oil were positioned in two different flasks. Airflow from the pump (NUP-1, AS ONE, Osaka, Japan) was switched on using an electro-magnetic bulb (VDW350-6G-2-01-A, SMC, Tokyo, Japan) between the two flasks to allow air to enter the tunnel through the pipes with or without any odor.

Before the experiments, airflow was observed by generating smoke inside the tunnel to identify the odor spread area [16]. The floor and walls were covered by black paper to create a contrast to the smoke during this measurement. The smoke image was extracted by subtracting the background from the original image for each movie frame. The odor spread area was determined to be the region estimated by superimposing the smoke images.

### 2.4. Measurements

All the experiments were performed from September to November, 2009, at the Himeji-Kankyoningen campus, University of Hyogo, Himeji, Japan. The video recording was started after removing every bee and food plate from the tunnel. Before the session of recording, the inside of the tunnel was cleaned by cloth impregnated with anhydrous ethanol (99.5%) to remove the odors that bees potentially deposited there. When one foraging bee entered the tunnel through the entrance hole, the hole was closed to prevent any interaction between individual bees. The air with or without odor stimulus was presented 5 s after the bee entered. Basically, recording was continued for 1 min, but was stopped if the bee landed on the floor for several reasons, such as being exhausted or overwhelmed. The recorded bee was removed from the tunnel and was not used again for the following experiments. After the end of each measurement, the food plate was repositioned for at least 15 min to revisit the food source and maintain foraging motivation. 

### 2.5. Data Analysis

The video data was transformed into a sequence of bitmap image files (BMP format) using Virtual DubMod [19]. The honeybee image was extracted from the original image by subtracting the background image. The center of gravity of the extracted region was calculated as the representative point on the honeybee’s body. A flight trajectory was obtained by connecting the representative point in each image frame and smoothed using a 5-point moving average filter. All of the data processing was done in MATLAB (MathWorks, Natick, MA, USA).

To investigate the effect of odor on a honeybee’s position in the tunnel, the remaining time rate in the tunnel was plotted. The remaining times with and without the odor, within a 15 cm radius of the odor stimulus pipe, were calculated and compared using the Mann-Whitney U test. When the odor was presented, bees took from 2 to 20 s to arrive at the odor pipe. We analyzed the remaining times separately in three groups: arrival at the pipe within 3 s (Under3), 10 s (Under10) and greater than 10 s (Over10).

As with other behavioral indexes, the flight speed over the ground, *ν_ground_* (m/s), upwind flight speed, *ν_upwind_* (m/s), and angular velocity, *ω* (°/s), were calculated from the flight trajectory.


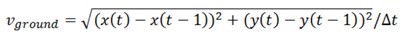
(1)


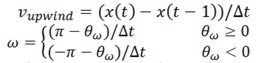
(2)


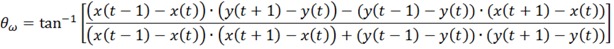
(3)

Here, *x* and *y* show the coordinate values in the direction of the long and short axes of the tunnel. *t* is the time and ∆*t* is the sampling period, 1/30 s. In the Under3 group, behavioral changes before and after stimulation were investigated using these indexes. These values were calculated and tested by the Friedman test for every second before and after 3 s from presenting the stimulus. Trajectories to the odor source were also investigated for 3 s before arrival at the pipe. Flight directions to the odor pipe along time were calculated frame-by-frame in this period. Behavioral indexes inside and outside the odor area were compared using the Mann-Whitney U test. 

## 3. Results and Discussion

### 3.1. Results

#### 3.1.1. Searching Area

**Figure 2:**
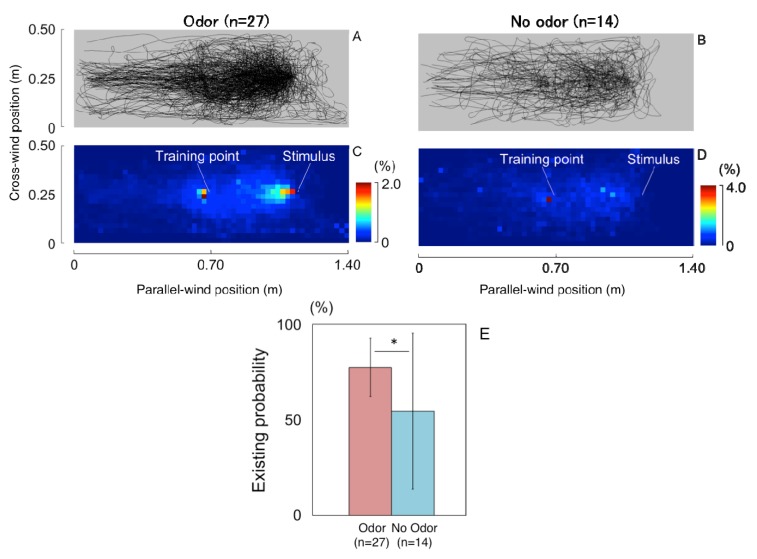
The flight trajectories and the remaining time rate in the tunnel. (**A**) The flight trajectories during odor stimulation. (**B**) The flight trajectories without odor stimulation. (**C**) The remaining time rate map in the tunnel during odor stimulation. Two peaks at the training and stimulus points are observed in this case. (**D**) The remaining time rate map in the tunnel without odor stimulation. The trajectories were distributed over a wide area, and only one peak was observed at the training point. The maps had a resolution of 56 × 20 pixels, and each side was 2.5 cm long. (**E**) The difference in the remaining percentage between odor and non-odor air injections at the back (upwind) of the training point (* *p* < 0.05, Mann-Whitney U).

It was established that the search area during a foraging flight was changed by the odor stimulus. There were two peaks around the feeder position and the odor pipe (Figure 2A,B). However, when we provided air with no odor, the bee searched the whole tunnel, particularly around the trained position for foraging (Figure 2C,D). By comparing the remaining time rate within a 15-cm radius of the stimulus pipe, a significant difference was shown as a result of the odor stimulus (Figure 2E) (*p* < 0.05, Mann-Whitney U).

**Figure 3:**
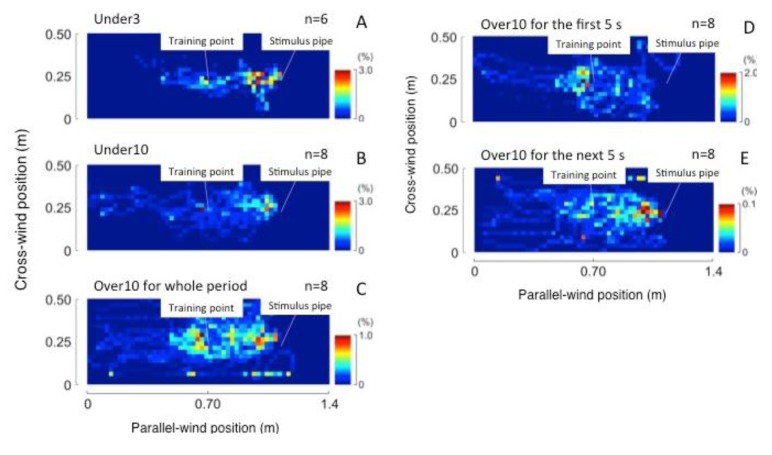
The remaining time rate for the three groups, arrival at the pipe within 3 s (Under3), 10 s (Under10) and greater than 10 s (Over10). (**A**) Under3, (**B**) Under10, (**C**) Over10 for the whole period. (**D**) Over10 for the first 5 s; (**E**) Over10 for the next 5 s.

The remaining time rates with odor stimuli were plotted separately in three different groups. In the Under3 group, the search area was very narrow and straight along the wind flow in the lee of the pipe (Figure 3A). A wider area was searched, and another peak at the training point appeared in the Under10 group (Figure 3B). It was shown that bees searched both of the surrounding areas of the training and stimulus points in the Over10 group (Figure 3C). Their flight area was wider than the other two groups. It is interesting to see that they searched around the training points in the first 5 s, then moved to the odor pipe (Figure 3D,E). 

#### 3.1.2. Flight Properties

**Figure 4:**
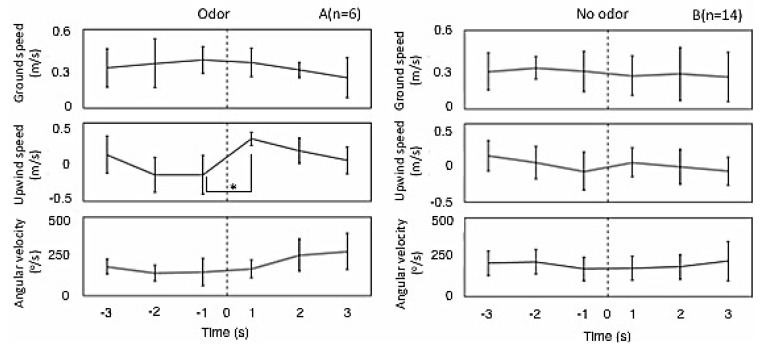
Flight properties of the ground speed, upwind speed and angular velocity for Under3. (**A**) Presenting air with odor (* *p* < 0.05, Friedman); (**B**) presenting air without odor. Graph points are the average values, and error bars give the standard deviation over 1 s. The dotted line shows the time of applying the odor stimulus.

We analyzed changes in the flight properties in the Under3 group, which exhibited a quick response to the odor stimulus when it was presented (Figure 4A). The ground speed decreased when presenting air with odor. The upwind speed gradually decreased with time, but significantly increased when odor was presented (*p* < 0.05, Friedman). Little effect from odor stimulus was observed on the angular velocity, but it did slightly increase with time. These changes were not observed when presenting air without odor (Figure 4B).

**Figure 5:**
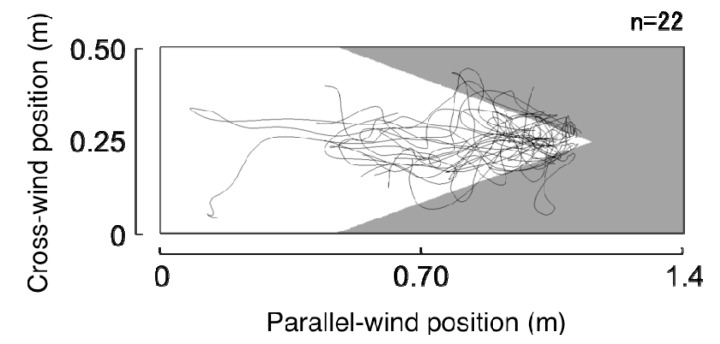
Flight trajectories for the 3 s before arriving at the odor source. White shows the odor spread area, and grey is the non-odor area.

**Figure 6:**
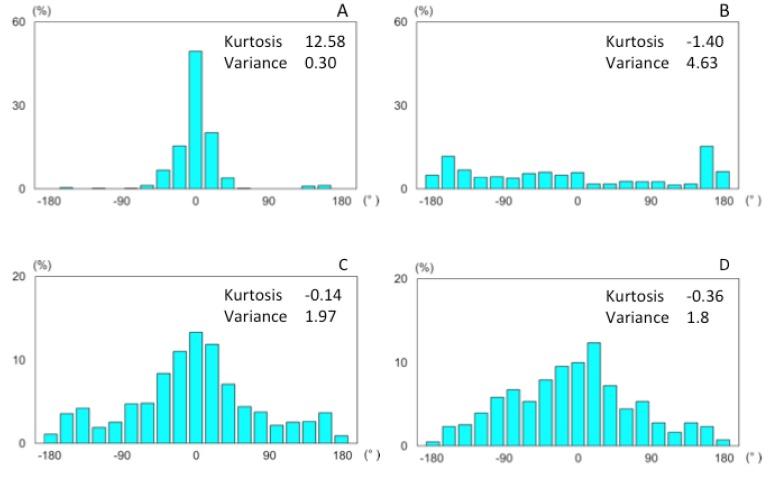
The histograms of flight direction to the odor pipe (n = 22): (**A**) during one second after entering the tunnel; (**B**) during one second before presenting the odor stimulus; (**C**) inside of the odor-distributed area during the presentation of the odor stimulus; and (**D**) outside of the odor-distributed area during the presentation of odor stimulus.

The flight trajectories just before arriving at the odor pipe were analyzed for 22 bees. Five samples in the total odor stimulated trials could not be included, because they could not reach there. The flight course in the tunnel was relatively straight near the entrance, but crosswind movements were increased as they approached the odor pipe (Figure 5). Furthermore, the search area reduced as the bees approached the odor source, but slightly extended beyond the border of the odor spread area. It was also seen that flight directions were changed by different situations. Bees flew into the tunnel and advanced into the back straightly (Figure 6A). However, bees searched around the feeder, and flight directions were distributed widely (Figure 6B). By presenting the odor stimulus, they flew toward the odor source both inside and outside of the odor-distributed area (Figure 6C,D)

**Figure 7:**
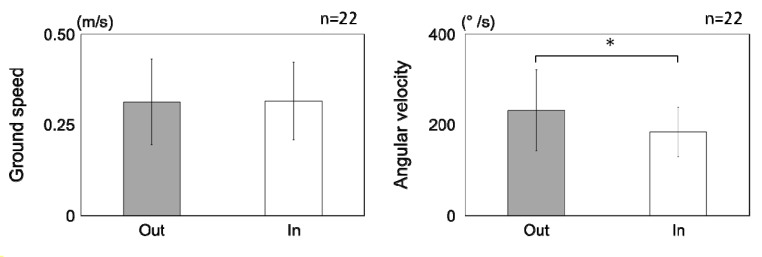
Comparison of ground speed and angular velocity between the inside and outside of the odor spread area for the last three seconds before arriving at the odor source. Bars show the average values and error bars the standard deviation (* *p* < 0.05, Mann-Whitney U).

There was no difference in ground speed between the inside or outside of the odor spreading area, but a significant difference was observed in angular velocity (Figure 7). This implies that rapid directional changes occurred outside the border of the odor area.

### 3.2. Discussion

#### 3.2.1. Search Area

It was established that the honeybee changed its flight behavior while approaching an odor source associated with food. We changed the position of the odor source in the test phase from that in the training phase. Our experimental conditions corresponded to the natural situation where a forager often visits her memorized foraging place again, but searches another place containing another key stimulus, such as an odor, when the memorized place no longer contains any nectar.

It was confirmed that foragers remembered and searched their foraging place carefully in any condition, with or without odor. During a foraging flight, a honeybee cannot only remember and use the odor of food, but also information from several flight routes, such as the direction and distance from her hive and site information and the color and shape of the nectar source [20,21,22,23,24,25,26,27,28,29]. We painted the inside of the tunnel white to avoid any effect on the visual information, but still retained several visual cues, such as a stain on the wall, fans for generating wind flow and the video camera, to aid in the bee’s return to the training point without the odor being present.

However, a bee could reach another point only with odor stimulus. Flight trajectories in this case covered the leeward area of the odor source. The changes in properties in these cases could be caused by differences in information for flight control; that is, distance and directional information were used in returning to the training point, but approaching the odor flow was achieved only by following odor plumes. The flight route without the odor source is smooth without turning and rotation [30]. In contrast, flights guided by odor were complicated, with repeated turns. Our results demonstrated that orientation flight was induced both inside and outside of the odor spread area (Figure 6); on the other hand, the angular velocity outside of the odor spread area is significantly larger than that inside of the odor spread area (Figure 7). This suggests that, at the edge of the odor spread area, the turns and rotations might frequently occur for orientation toward the odor source. Chaffiol *et al*. suggested that orientation flight (upwind zigzag flight) and circling behavior toward the learned odor-source were significantly induced by the prior olfactory conditioning by using proboscis extension reflex (PER) [31]. The odor-guided honeybees detect the edge of the odor spread area during these orientated flight and circling for the local search of the odor source.

Times for arriving at the odor pipe after the stimulus was turned on had a large variance for the Under3 to the Over10 groups. Honeybees in the Under3 group could recall the odor soon and switched their flight direction to the odor source. The Over3 and Over10 groups were fixated on the learning point, even with no food or odor source present; then, they changed their behavior to seek a real odor source later. The flight to the odor source in our experiments might correspond to the odor recall flight, which was shown by the injection of the previously visited feeder’s odor [32].

Several reasons are considered for the behavioral differences between individual bees. One could be the difference in the ages of the bees, affecting learning and memory capabilities [33,34]. The experience of foraging could also have an effect on their foraging properties. As a next step in our experiment, it is important to control the age and experience of individual bees by identifying the marks on individuals.

#### 3.2.2. Behavioral Properties

The upwind speed was increased by the odor stimulus, but not the ground speed (Figure 4). This implies that the percentage of flights to the odor source increased. Other insects can search the odor source from upwind. The increase in upwind speed just after the odor stimulus was the same for *Drosophila* as in our results [13].

The flight area near the odor pipe was coincident with the distribution area of the odor. This was caused by the drastic change in flight direction when they left a non-odor area. This is supported by the increase in angular velocity just outside the odor spread area. Characteristic behaviors, zigzags or turns caused by losing the odor stimulus are observed in moth, but not in honeybee, flight. It might be that there are differences in the flight environments and the control mechanisms between moths and honeybees. The honeybee mainly uses visual information for flight control and navigation, as do other diurnal insects. The honeybee might use odor information only for orientation to the odor source, whereas most moths use odor information for nocturnal flights. Furthermore, the difference in flutter speed could reflect differences in the sensory-control mechanisms between bees and moths and the flight trajectories of these insects; that of a moth is about 30 Hz [35,36], and that of a honeybee is over 200 Hz [37,38]. In this study, we investigated flight properties for only one condition. It is important to investigate, in more detail, flight properties under multi-modal stimuli and complex environmental conditions. 

## 4. Conclusions

We observed honeybee flight approaching an odor source in a wind tunnel. We separated the bees’ memory-guided and odor-searching flights by changing the position of the odor stimulus in the training and test sessions. When presented with an odor stimulus, some individual bees quickly changed from their memory-guided flight to an odor-searching flight. However, others took more time to reach their destination. They recalled memorized places and approached them first, then changed their behaviors to search for an odor source. We could not clarify the reason for these individual differences, but the variety in individual behavior might be important for maintaining effective foraging activities in a hive, even under various environmental conditions. In a honeybee, quick directional changes in flight were observed just outside an odor spread area, but we did not observe a zigzag flight pattern as in a moth. This could reflect the difference in the sensory-control mechanisms between bees and moths. We used only a single bee colony in our experiment. Future studies with additional colonies would be desirable to replicate our results.
